# Transcriptional responses of *Eucalyptus* to infection by an aggressive leaf blight pathogen reveal the role of host secondary metabolites during pathogen germination

**DOI:** 10.1007/s11103-025-01625-2

**Published:** 2025-08-11

**Authors:** Myriam Solís, Almuth Hammerbacher, Michael J. Wingfield, Sanushka Naidoo

**Affiliations:** 1 Department of Biochemistry, Genetics and Microbiology, Forestry and Agricultural Biotechnology Institute (FABI), University of Pretoria, Pretoria, 0002 South Africa; 2 Department of Zoology and Entomology, Forestry and Agricultural Biotechnology Institute (FABI), University of Pretoria, Pretoria, 0002 South Africa

**Keywords:** RNA-seq, Eucalyptus, Metabolites, Leaf pathogen, *Teratosphaeria destructans*

## Abstract

Teratosphaeria leaf blight disease caused by *Teratosphaeria destructans* poses a serious threat to *Eucalyptus* plantations worldwide. The pathogen infects leaves via stomatal penetration from 24 to 72 h after inoculation. Symptoms are visible after two weeks and pathogen sporulation commonly occurs four weeks after inoculation of a susceptible host. We studied the responses of a susceptible *Eucalyptus* clone during the entire disease cycle to identify susceptibility factors. RNA from healthy and infected leaves was isolated at 3, 14 and 28 days post inoculation. Differential expression and gene enrichment analysis showed that members of the transcription factor family *TGA* and *MYB,* involved in the salicylic acid and abscisic acid pathways, and genes involved in these hormone signaling pathways, were up-regulated. Overall, plant defense response pathways were enriched only at the late stage of infection (28 dpi). In contrast, both gene expression and chemical analysis revealed that the synthesis of the major flavonoids in Eucalyptus leaves was enhanced during pathogen infection, while the synthesis of terpenoids and flavan-3-ols declined. The flavonols, rutin and quercetin enhanced spore germination in-vitro while, the terpenoid eucalyptol and the flavan-3-ol catechin inhibited germination. This study provides insights into the molecular and chemical responses at different stages of infection of a susceptible host by *T. destructans*, thereby improving the current understanding of the pathosystem.

**Supplementary Information:**

The online version contains supplementary material available at 10.1007/s11103-025-01625-2.

## Key message

A susceptible *Eucalyptus* host to the aggressive leaf pathogen *Teratosphaeria destructans* showed a delay in the plant immune response during pathogen infection. The transcriptome and metabolomic analysis revealed that flavonoid and terpenoids are able to modulate pathogen germination in-vitro.

**Supplementary Information:**

The online version contains supplementary material available at 10.1007/s11103-025-01625-2.

## Introduction

Teratosphaeria leaf disease is one of the most common foliar diseases of *Eucalyptus* (Coutinho et al. 1998; Crous et al. 2007; Hunter et al. 2011). The majority of the *Teratopshaeria* species causing this disease are weak pathogens (Maxwell et al. 2003; Hunter et al. 2011; Crous et al. 2019). However, within this group of closely related species, a few aggressive pathogens cause severe disease known as Teratosphaeria leaf blight (TLB) (Carnegie and Ades 2003; Andjic et al. 2019; Crous et al. 2019). TLB symptoms are characterized by leaf chlorosis and shoot malformation (Andjic et al. 2019). In temperate areas, TLB is commonly caused by *Teratosphaeria nubilosa* and *T. cryptica* (Mohammed et al. 2003; Hunter et al. 2011; Burgess and Wingfield 2017), whereas in tropical and sub-tropical plantations the disease is caused by *T. destructans*, *T. eucalypti*, *T. pseudoeucalypti*, and *T. viscida* (Andjic et al. 2007, 2010, 2011; Crous et al. 2019).

Amongst *Teratosphaeria* species *T. destructans* is particularly aggressive and has rapidly spread throughout tropical and subtropical *Eucalyptus* plantations worldwide (Wingfield et al. 1996; Old et al. 2003; Burgess et al. 2006; Andjic et al. 2011; Barber et al. 2012; Greyling et al. 2016; Havenga et al. 2021a, b). The pathogen mainly affects *Eucalyptus grandis* and its hybrids, the most widely planted *Eucalyptus* species for industrial wood production (Cunningham and Bijay Tamang 2014). A study on a susceptible *E. grandis* × *E. urophylla* host showed that infection by *T. destructans* occurs by penetration through the stomata approximately 48 h after inoculation (Solís et al. 2022). The growth of the pathogen occurs intercellularly and the characteristic leaf discoloration or deformation symptoms appear in susceptible hosts approximately two weeks after inoculation. Following symptom development, spores are released through the stomata, predominantly on the abaxial leaf surface (Solís et al. 2022).

Host susceptibility is typically defined from the perspective of a loss of resistance (Chisholm et al. 2006). Plant susceptibility gene (S-gene) products can provide signals that attract pathogens and increase their aggressiveness (Eckardt 2002; Lapin and Van den Ackerveken 2013). For example, the S-gene, *Citrus sinensis lateral organ boundary 1 (CsLOB1)* allows for a compatible interaction between the host and *Xanthomonas citri* ssp. citri, causing citrus canker. Gene editing creating a loss of function of this gene resulted in host resistance to this pathogen (Jia et al. 2017; Engelhardt et al. 2018). The latter example emphasizes the need to understand host susceptibility mechanisms, in addition to resistance mechanisms for crop improvement.

Plant resistance is commonly related to a suite of plant immune responses to pathogen invasion that include pathogen recognition, signal transduction, and the expression of products that allow the host to interact directly with the pathogen (Jones and Dangl 2006). The early activation of transcription factors (TFs) related to phytohormone defense pathways is known to be a key factor in the success of early pathogen recognition (Alves et al. 2014; Ng et al. 2018). Previous studies have shown that early activation of hormone signaling, such as salicylic acid (SA), jasmonic acid (JA), and ethylene (ET) are often activated during plant pathogen infection (Robert-Seilaniantz et al. 2011; Kazan and Lyons 2014; Solís et al. 2022). Downstream signaling networks initiate multiple responses, including the biosynthesis of secondary metabolites (Lv et al. 2021). These metabolites are among the most important host defense responses which non-specifically target a wide variety of pathogens (Kliebenstein 2004).

The principal secondary metabolites produced by *Eucalyptus* are phenolics and terpenoids (Brezáni and Šmejkal 2006). Phenolic compounds are well-known plant defenses and some have been shown to have strong antimicrobial properties (Ullah et al. 2017, 2019; Hammerbacher et al. 2019a, b; Kumar et al. 2020). In addition, eucalyptol (1,8-cineole), the major terpenoid produced in the foliar oil glands of *Eucalyptus*, can inhibit fungal growth of some plant pathogenic fungi (Morcia et al. 2012). Despite being an important pathogen of *Eucalyptus,* little is known about the molecular mechanisms involved in the host interaction with *T. destructans.* To facilitate molecular and biochemical studies, we established an artificial inoculation protocol under controlled conditions for *T. destructans* (Solís et al. 2022), which can be used to elucidate the responses that underlie host symptoms. Using this approach, the aim of this study was to elucidate the main pathways and genes activated by *T. destructans* in a susceptible *E. grandis* × *E. urophylla* genotype during the infection cycle. In addition, the effect of plant secondary metabolites, produced by *Eucalyptus* during pathogen infection, on spore germination was assessed.

## Methods

### Plant material and inoculation

One-year-old *Eucalyptus grandis* × *E. urophylla* ramets of a single clone were used in this study. This clone is known to be highly susceptible to infection by *T. destructans* (Greyling, Mondi Forests South Africa, personal communication). Plants were maintained in 5 L black polyethylene bags containing potting soil mix, under natural light and watered twice per day. The daytime temperature ranged from 20 to 25 °C, with an average night temperature of 20 °C during the three-months, before experiments were conducted.

Twenty-four healthy plants were selected for the study, twelve of which were inoculated with a conidial suspension of *T. destructans* isolate CMW56797 (Culture collection of the Forestry and Agricultural Biotechnology Institute, University of Pretoria) taken from three-week-old cultures, maintained at 25 °C. The conidial suspension was prepared by washing the cultures with sterile distilled water + 0.01% of Tween 20 (Sigma-Aldrich). The concentration was adjusted to 1 × 10^6^ conidia/ml using a hemocytometer. Plants were inoculated on both leaf surfaces by spraying the leaves with the spore suspension until runoff. Subsequently, plants were enclosed with clear plastic bags to maintain high humidity levels for 72 h. The twelve control plants were sprayed with sterile distilled water to which 0.01% of Tween 20 (Sigma-Aldrich) was added and otherwise treated in the same manner as the inoculated plants.

**Fig. 1:**
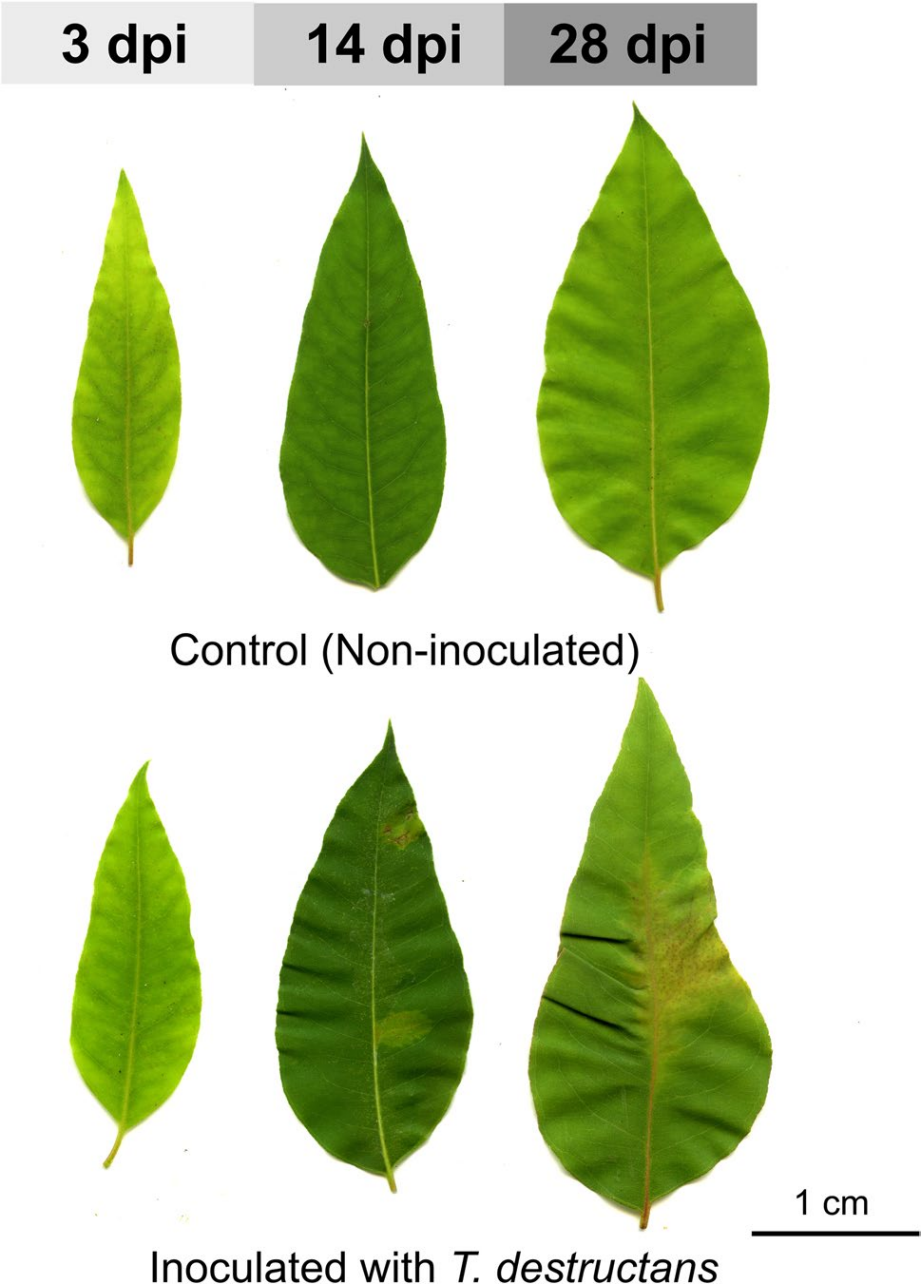
Disease progression of *Eucalyptus grandis* ×* E. urophylla* leaves infected with *Teratosphaeria destructans* at 3, 14 and 28 days post inoculation (dpi). The top leaves show the control at different time points and the bottom leaves are inoculated with *T. destructans.* Symptoms appeared from 14 dpi and became more evident at 28 dpi. Scale bar 1 cm

After removing the plastic bags, the plants were maintained in a greenhouse under natural light with a day temperature ranging from 20 to 25 °C and a night temperature of 20 °C. Twelve juvenile expanded leaves per plant were harvested from the inoculated and control plants, at 3 (early infection), 14 (colonization) and 28 (late infection) days post inoculation (dpi) (Fig. 1). Four replicates were harvested for each time point from the control and inoculated plants. The samples were maintained at − 80 °C for RNA extraction, RNA-seq and metabolite analysis.

### RNA extraction and sequencing

Total RNA was extracted from four leaves from each of the four non-inoculated and inoculated replicates at 3, 14 and 28 dpi using the Maxwell 16 LEV simplyRNA Tissue Kit (Promega, USA). RNA extraction, library preparation and next-generation sequencing was performed by Macrogen, Korea. Extracted RNA was assessed using an Agilent Technologies 2100 Bioanalyzer (2200 TapeStation) to ensure sample integrity (RNA Integrity Number > 7.0) and purity (absence of genomic DNA). The SMARTer smRNA-Seq Kit (Takara Bio Inc., USA) was used for library preparation, and Illumina Novaseq 6000 S4, 100 bp pair-end configuration with 40 million reads per lane, was used for sequencing. The sequencing data was delivered as raw data FASTQ files.

### Mapping and gene expression analysis

The read quality from the RNA-seq data was analyzed using FASTQC v0.11.3. Reads from the control and inoculated leaves at each time point were mapped to the *E. grandis* v2.0 reference genome (Myburg et al. 2014) using STAR (Dobin et al. 2013). The mapped reads were normalized and variance stabilized transformation was applied using DESeq2 (Love et al. 2014) and transcripts per million kilobases (TPM) was calculated in R v3.6.0 (R Core Team 2018). The differentially expressed genes (DEGs) were statistically analyzed using DESeq2 with a cut-off of $\ge$ 1.00 $\le$ – 1.00 log_2_ (fold change) and a false discovery rate (FDR) of *p* < *0.05*. The changes in gene expression were compared to control plants derived from uninfected tissue at each time point.

Gene Ontology (GO) enrichment analysis was implemented (Young et al. 2012) and Kyoto Encyclopedia of Genes and Genomes (KEGG) (Kanehisa and Goto 2000) annotation enrichment of the DEGs was performed to identify biological pathways potentially involved at each time point of interaction. In addition, enrichment of transcription factor (TF) families was also performed as described by Oates et al. (2021), DEG’s were classified as TFs in the Plant Transcription Factor Database v4.0 (Jin et al. 2014, 2015, 2017) and grouped by family as defined by their associated *Arabidopsis thaliana* orthologues. Enrichment was calculated using Fisher’s exact test and *p-values* were adjusted using the Benjamini and Hochberg (BH) method with a FDR of *p* < *0.05*. The analysis was performed in R v3.6.0. Heatmaps were generated using the package Seaborn in Matplotlib library built on Python version 3.8.8 (2021) using log_2_ (fold change) values.

### LC–MS and GC–MS analysis for flavonoid and terpenoid identification

To study the secondary metabolites, flavonoid analyses were performed using liquid chromatography–mass spectrometry (LC–MS) and terpenoids by using gas chromatography–mass spectrometry (GC–MS). Four leaves each for the controls and inoculated plants at 3 and 28 dpi, respectively were used for each analysis. Four replicates per treatment were used for LC–MS and GC–MS analysis.

#### LC–MS analysis of flavonoids in leaves

For LC–MS analysis, a total of 20 mg ground leaves per sample were used for flavonoid extraction using 1 ml methanol (Sigma) in a 1.5 ml Eppendorf tube. The mix was incubated at 200 rpm at 24 °C for 1 h, followed by a centrifugation step at 1200 rpm for 20 min. Subsequently, 800 μl of the supernatant was transferred to a glass vial. A volume of 1 μl of the supernatant was analyzed using a Waters Acquity Ultra Performance Liquid Chromatography (UPLC®) system with ultra-pure LC-grade water and acetonitrile (Romil-UpS™, Microsep, South Africa) acidified with 0.1% formic acid (99 + % purity) (Thermo Scientific, South Africa). Compounds were eluted from a Luna® Omega 1.6 µm C18 100 Å (2.1 mm ID × 100 mm length) column using a 17 min gradient from 97% H_2_O to 100% acetonitrile followed by an isocratic wash step with 100% acetonitrile for 1 min and a column reconditioning step with 97% water for 2 min. Volumes of 7.5 µl were injected onto the column heated to 40 ºC at a flow rate of 0.4 ml/min. The UPLC was coupled to a Waters® Synapt G2 high-definition quadrupole-time-of-flight (QTOF) mass spectrometer (Waters Inc., Milford, Massachusetts, USA) operated in negative ionization mode. The capillary voltage was 2.6 kV. Collision-induced fragmentation was performed at 4 V for the trap collision energy and the transfer collision energy was ramped from 20 to 40 V. Mass spectral scan intensities were collected every 0.3 s from 50 to 1 200 Da. MassLynx™ (version 4.1) software (Waters) was used for data acquisition and analysis. Relative quantities are reported based on peak areas.

#### GC–MS analysis of terpenoids in leaves

For GC–MS, 20 mg of ground leaves per treatment with four replicates, was aliquoted into a 1.5 ml Eppendorf tube and extraction was carried out using 1 ml hexane (Sigma), amended with 0.4 μl/ml of the internal standard, 2-phenylethanol. The mixture was incubated at 200 rpm at 24 °C for 1 h, followed by centrifugation at 1200 rpm for 20 min. 800 μl of the supernatant was transferred into a glass vial and 1 μl of the supernatant was used for the analysis using an Agilent 7890 GC–MS using an HP5 column (Agilent) with a linear temperature program starting at 40 °C increasing at a rate of 4 °C until a maximum temperature of 200 °C, then held for 2 min. The parameters of the GC–MS were a solvent delay of 3.5 min, split-less inlet and a flow rate of 1.2 ml/min. The mass spectrometer was set to scan mode with a low mass of 40 m/z and a high mass of 450 m/z and the ion source was maintained at 70 eV. The extracted chromatograms were analyzed using Agilent MassHunter® Qualitative Analysis software, build 8.0.598.0. Compounds were tentatively identified utilizing the 2017 NIST library (Information Services Office). MetaboAnalyst 4.0. was used for data exploration. Only relative quantities are reported based on peak areas.

### Effects of flavonoids and eucalyptol on pathogen germination

A bioassay to evaluate the effect of secondary metabolites on *T. destructans* conidial germination was performed using commercially available pure flavonoids and terpenoids, including eucalyptol, quercetin, rutin and catechin (Sigma-Aldrich, South Africa).

The effect of the compounds, eucalyptol, quercetin, rutin and catechin on germination was evaluated at six different concentrations; 0 μg/ml, 2 μg/ml, 10 μg/ml, 50 μg/ml, 200 μg/ml, 1 mg/ml. Each compound was diluted in ethanol (JT Baker). For each compound and concentration, 1 ml solution was distributed on the surface of a 50 mm diameter Petri dish and allowed to dry in a laminar flow cabinet. Subsequently, a volume of 150 μl of a *T. destructans* (CMW56797) conidial suspension was placed onto the surface of the Petri dish. The suspension was prepared as described above, and adjusted to 1 × 10^6^ conidia/ml (spores/ml). The Petri dishes with the different concentrations of each compound and conidia to be tested were placed in plastic boxes on a rack suspended 5 cm above sterile distilled water for 72 h to maintain a high humidity and maintained at 25 ºC. Four replicates were used per treatment. The effect on germination was assessed by counting the number of germinated conidia per 100 plated spores under a light microscope at 20× magnification using an Axioskop 2 plus microscope (Zeiss, Oberkochen, Germany).

### Statistical analyses

A completely randomized design was used for all the experiments. Statistical analyses and data visualization were performed using R version 3.2.0 (R Foundation for Statistical Computing, Vienna, Austria). Data were normalized using square-root transformations. The data were analyzed statistically for GC–MS, LC–MS and in-vitro assays using ANOVA and Tukey’s post-hoc test was used to determine the significance of differences between all treatments (*p* < *0.05*). Graphs were created using ggplot2 (Wickham 2009) in R version 3.2.0.

## Results

### Differential gene expression

**Fig. 2:**
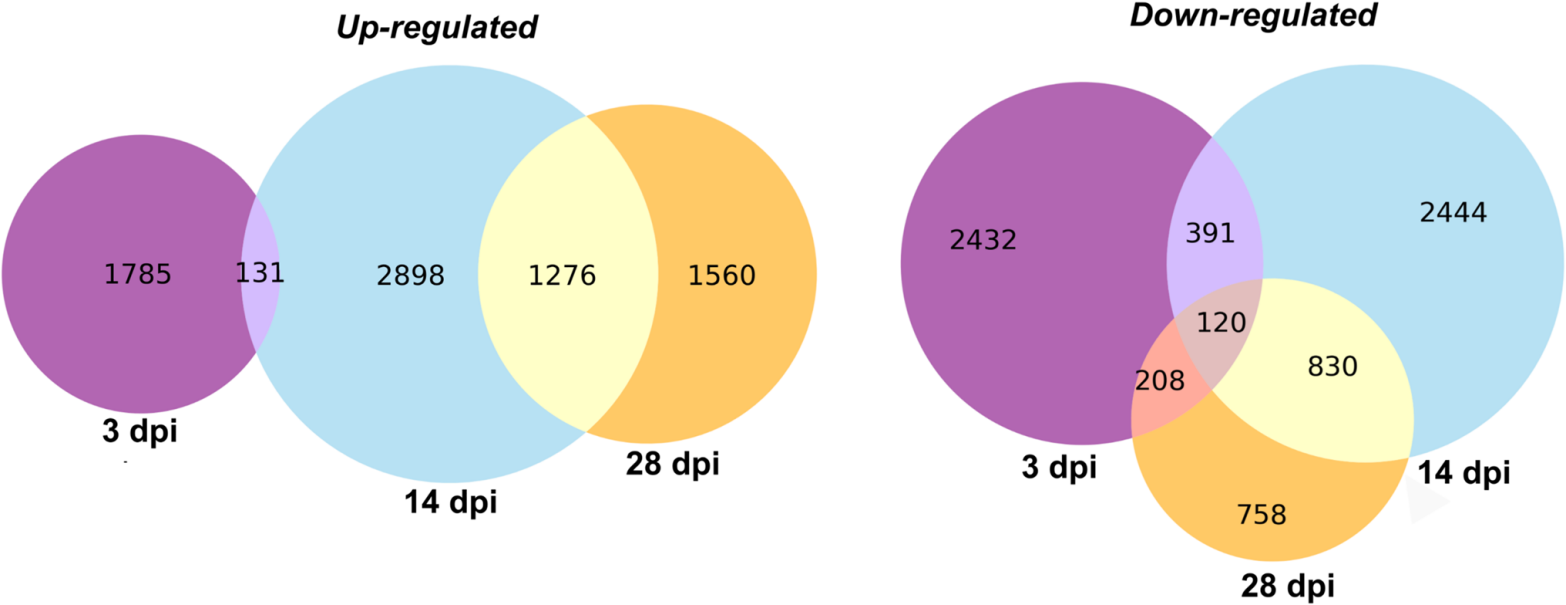
Venn diagram of up-regulated and down-regulated differentially expressed genes (DEGs) identified through RNA-seq analysis of the leaves of a susceptible *Eucalyptus grandis* ×* E. urophylla* host during infection with *Teratosphaeria destructans*, at 3 days post inoculation (dpi), 14 dpi and 28 dpi. Up-regulated genes had a log_2_ fold change $\ge$ 1.00 while genes with $\le$ − 1.00 log_2_ were considered down-regulated

The differential global expression analysis considering the three different time points, 3, 14 and 28 dpi compared with their respective non-inoculated control plants revealed 7740 up-regulated and 7183 down-regulated DEGs at all the time points (Fig. 2). Up-regulated genes were not shared between extreme time points (3 and 28 dpi), an expected result, considering the influence of the time on leaf development, as well as the stage of the pathogen’s infection (Fig. 2).

**Fig. 3:**
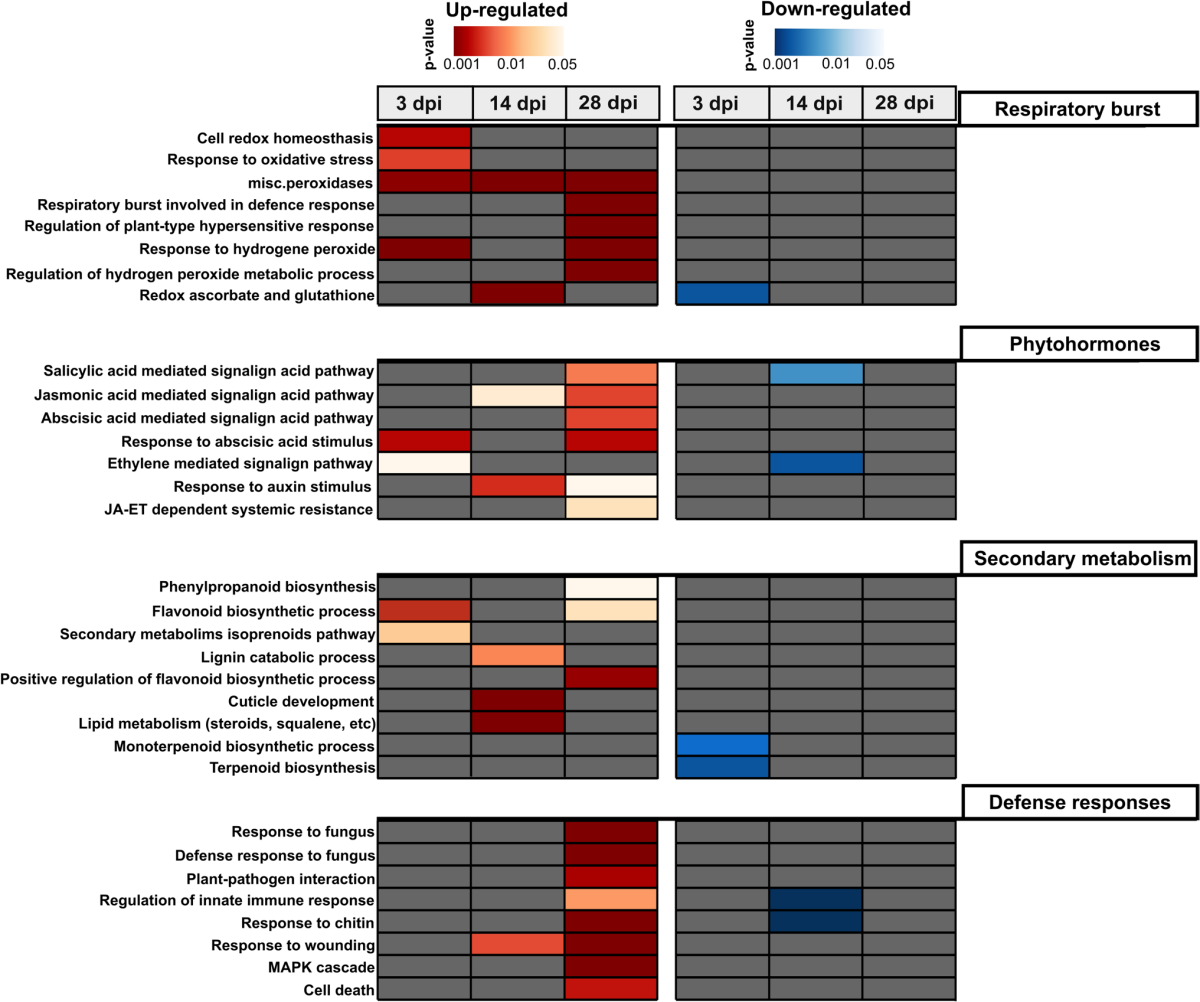
Heatmap of the main pathways identified by gene ontology (GO) enrichment analysis of differentially expressed genes in leaves of a susceptible *Eucalyptus grandis* ×* E. urophylla* host inoculated with *T. destructans*, at 3 days post inoculation (dpi), 14 dpi and 28 dpi. Plant defense terms were classified into respiratory burst, phytohormone signaling, secondary metabolism and defense responses. Color bar indicates the significance (*p* < 0.05), grey blocks indicate the absence of the pathway on the enrichment analysis. Red represents up-regulated GO terms and blue represents down-regulated GO terms

During the early stage of the infection (3 dpi), most of the up-regulated genes performed functions related to peroxidases, transcription regulation and sugar transport (*p* < 0.05) (Supplementary material, Fig. 1). During host colonization at 14 dpi, the most significantly enriched genes were those involved in responses to wounding (*p* < *0.05*), whereas down- regulated genes were involved in protein phosphorylation, sugar transport and regulation of the hypersensitive response (Supplementary material, Fig. 1). At the latest stage of infection (28 dpi) when sporulation of the pathogen had occurred, a greater number of genes were up-regulated (2836) and they were mainly those involved in plant defense responses and oxidation reduction signaling (Fig. 3 and Supplementary Material, Fig. 1). A larger number of genes related to sugar and transmembrane transport were down-regulated at 28 dpi (Supplementary Material, Fig. 2), most probably due to tissue necrosis resulting from the infection.

The GO enrichment analysis showed that the most representative differentially expressed pathways involved in plant defense were classified into respiratory burst, phytohormone signaling and secondary metabolism (Fig. 3). Additionally, at each time point, up and down-regulated terms were categorized according to their significance (*p* < 0.05) (Fig. 2). At 3 dpi, the early stage of infection, the main enriched term was respiratory burst that involved the up- regulation of genes associated with a response to hydrogen peroxide (*p* < 0.001), cell redox homeostasis (*p* < 0.001), peroxidases (*p* < 0.001) and response to oxidative stress (*p* = 0.01). At 14 dpi, respiratory burst was only enriched for miscellaneous peroxidases and redox ascorbate and glutathione, involved in ROS detoxification. At 28 dpi most of the pathways related to respiratory burst were again significantly up-regulated (*p* < 0.001) (Fig. 3). These results demonstrate a delay in the activation of genes involved in the respiratory and oxidative burst, which were only activated at the late stage of infection by *T. destructans*.

### Enrichment of TFs and phytohormone signaling pathways

The main TFs differentially expressed across the time points were classified as *NAC, MYB, MYB_related, bHLH, ERF C2H2, WRKY* and *bZIP* (Supplementary material Fig. S2)*.* These TF’s were enriched at the latest stage of infection. During the early stage of infection, a homologue of *MYB96* (*Eucgr.B00502*) a regulator of ABA signaling was significatively up-regulated (*p* < 0.005) (Supplementary material Fig. S3) and the transcription of a *bZIP* TF homologue to *TGA10* (*Eucgr.K01822*) involved in regulating SA signaling increased at the late stage of infection (fold change = 9; *p* < 0.005) (Supplementary material Fig. S5). Consistent with these results, genes involved in the SA, JA, ET, ABA and auxin signaling pathways were significantly up-regulated at 28 dpi, while showing inconsistent responses at 3 and 14 dpi (*p* < 0.005) (Fig. 3). The delay in the defense response was even more evident when considering defense-related GO terms such as defense response to fungus, response to chitin, regulation of innate immune response, cell death and plant-pathogen interaction, which were all significantly up-regulated only at 28 dpi (Fig. 3). In contrast, the TF *ATAF1* a negative regulator of plant defense responses was up-regulated at the earlier and latest time points of infection (Supplementary material Fig. S6) which might play a critical role in the success of the pathogen infection.

### Expression of secondary metabolite biosynthesis genes

**Fig. 4:**
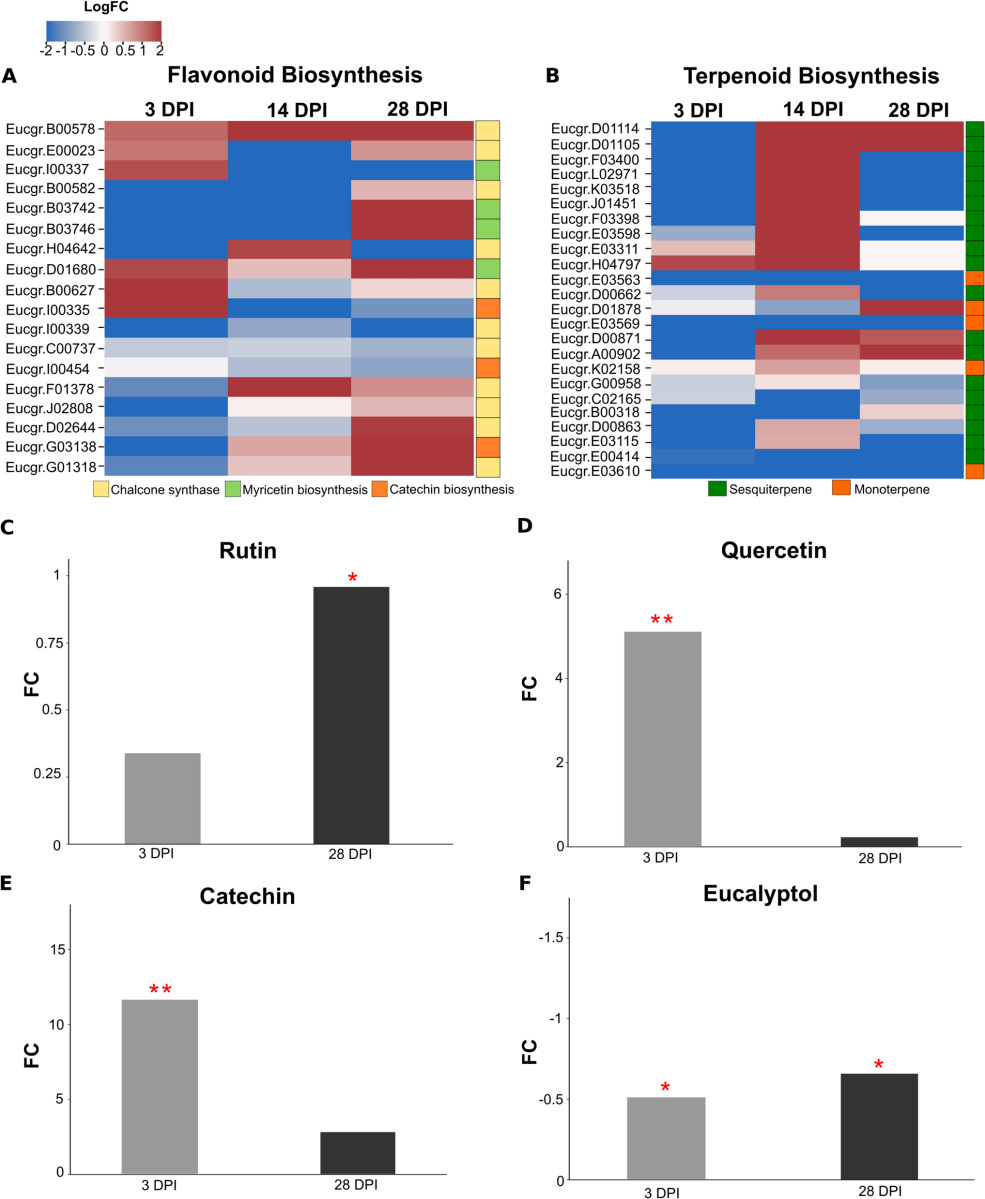
Differentially expressed genes involved in flavonoid and terpenoid biosynthesis and the abundance of the major compounds identified by LC–MS and GC–MS in *E. grandis* ×* E. urophylla* inoculated with *T. destructans* at 3 and 28 dpi. **A**, **B** Heatmap of the genes involved in flavonoid (**A**) and terpenoid (**B**) biosynthesis. Red and blue represent up- and down-regulated genes, respectively. Heatmap shows log_2_ fold change with *p* < *0.01*. **C**–**E.** Fold change (FC) values of the major flavonoids identified by LC–MS on *Eucalyptus grandis* ×* E. urophylla* leaves inoculated with *T. destructans* at 3 and 28 dpi compared to the abundance on the control leaves at each time point. **F** FC values of eucalyptol identified by GC–MS in *Eucalyptus grandis* ×* E. urophylla* leaves inoculated with *T. destructans* at 3 and 28 dpi compared to the abundance on the control leaves at each time point. Error bars indicate the standard error of the mean. Statistical differences are marked at the top of the bar plot with *(*p* ≤ *0.05*), **(*p* ≤ *0.01*) based on Tukey-test. N = 4

Gene enrichment analysis of secondary metabolic pathways showed an up-regulation of genes related to flavonoid biosynthesis in the initial phase (3 dpi) and at the later stage (28 dpi) of infection (Fig. 3). At 3 dpi, genes involved in terpenoid biosynthesis were significantly down-regulated, except for the up-stream methylerythritol phosphate pathway (secondary metabolism isoprenoid non-mevalonate pathway), which provides the precursors for monoterpene biosynthesis (Fig. 3). The DEG analysis of the genes related to flavonoid biosynthesis at each time point revealed that chalcone synthase and genes encoding downstream enzymes in the pathway were up-regulated in the leaves during *T. destructans* infection (Fig. 4A). Genes involved in sesquiterpene and monoterpene biosynthesis were, however, significantly down-regulated in the early and late stages of the infection process, and the more abundant transcripts were related to sesquiterpene synthase genes (Fig. 4B).

### Flavonoid and terpenoid accumulation

The flavonoids identified by retention time and mass spectra in the LC–MS analysis included rutin, quercetin and catechin (Fig. 4C–E). The flavonoids, quercetin and catechin were more abundant in young (3 dpi), freshly inoculated leaves, compared to the fully expanded leaves (28 dpi) (Fig. 4D, E). In contrast, rutin was significantly more abundant in the inoculated leaves at the later stage of infection (*p* < 0.01) (Fig. 4C). The GC–MS analysis enabled the identification of eucalyptol, based on retention time and pure standards, as the major terpenoid in the extract. Eucalyptol showed significantly lower abundance in leaves inoculated with *T. destructans* at 3 and 28 dpi compared to the controls (*p-value* < 0.001) (Fig. 4F).

### Effect of secondary metabolites on spore germination

**Fig. 5:**
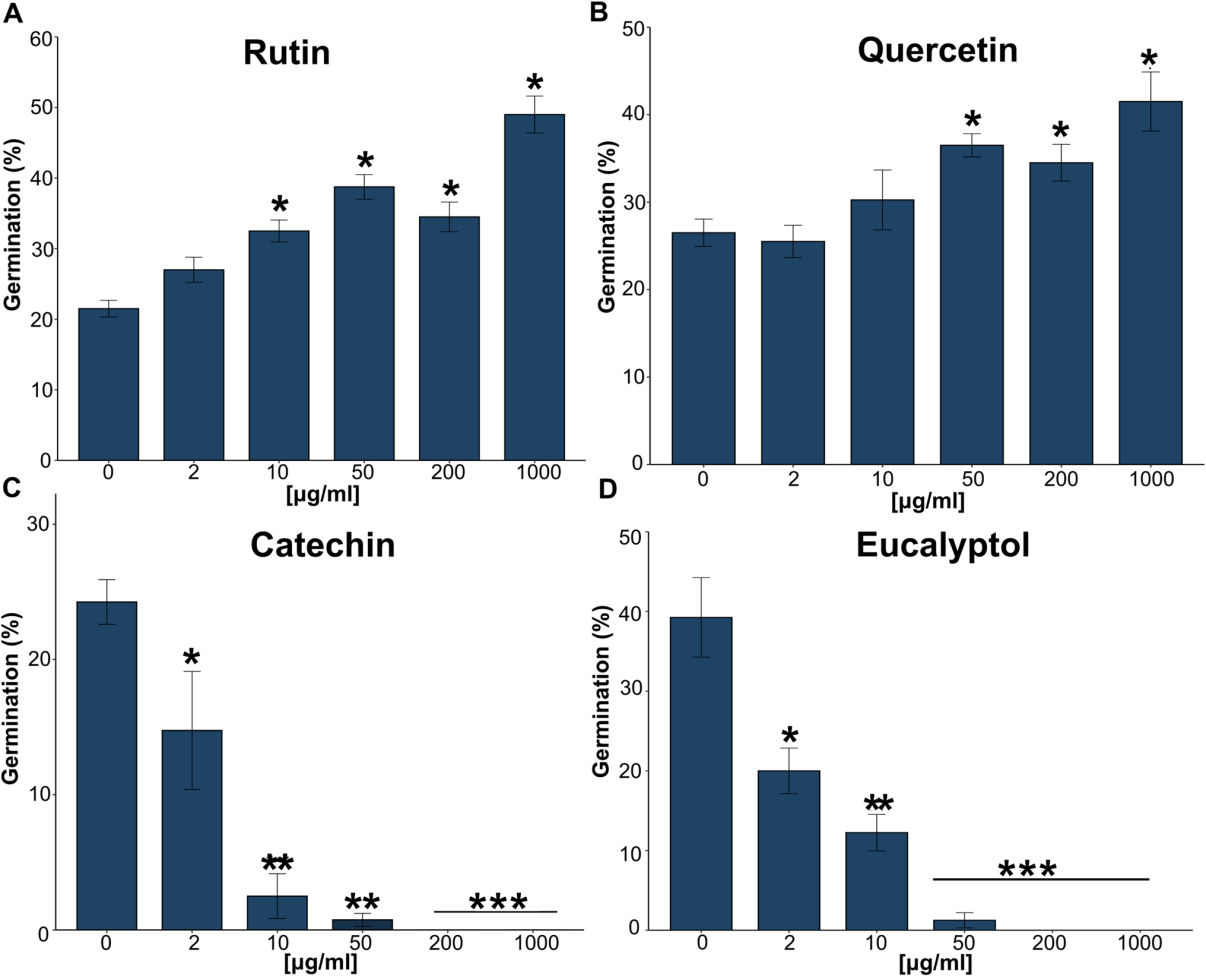
In-vitro effect of different concentrations of rutin, quercetin, catechin and eucalyptol on the germination of *T. destructans* at 72 h. **A**–**C** Effect on the germination of *T. destructans* by the flavonoids rutin, quercetin and catechin. **D** Effect on the germination of *T. destructans* by the terpenoid eucalyptol. Error bars indicate the standard error of the mean. Statistical differences are marked at the top of the barplot with *(*p* ≤ *0.05*), **(*p* ≤ *0.01*), ***(*p* ≤ *0.001*), based on Tukey-test. N = 4

The in-vitro assessment of the effect of the flavonoids on spore germination showed that rutin and quercetin significantly stimulated spore germination from approximately 20–30% germination (10 μg/ml) to 40–50% (50 μg/ml), respectively (*p* < 0.05) (Fig. 5A, B). In contrast, catechin significantly reduced the pathogen’s spore germination from 24% at the first concentration of 2 μg/ml to approximately 3% at 10 μg/ml of the compound (*p* < 0.01) (Fig. 5C). The different concentrations of the terpenoid, eucalyptol, also had a significant effect on *T. destructans* spore germination and reduced the germination from 38 to 20% from 0 to 2 μg/ml, with incremental decreases at higher concentrations (Fig. 5D). No spore germination was observed at the higher concentrations of 10 and 1000 μg/ml (*p*-value < 0.001) (Fig. 5D).

## Discussion

To gain a holistic view of the responses of a susceptible *Eucalyptus* genotype to *T. destructans* infection, differential gene expression and secondary metabolite accumulation in the host during different stages of infection cycle were analyzed. The results showed that most of the genes involved in host defense responses were expressed late (28 dpi) in the infection cycle. In contrast, flavonoid biosynthesis was up-regulated during early and late infection and these compounds stimulated spore germination. In contrast, terpenoids had a strong inhibitory effect on *T. destructans* spore germination and these genes related to this biosynthesis pathway were significantly down-regulated during the compatible interaction between the host and the pathogen.

At the early point of infection (3 dpi), genes related to sugar transport and peroxidase pathways were up-regulated. Sugars and peroxidases are known to enhance oxidative burst at early stages of infection, stimulating the synthesis of flavonoids and inducing the transcription of certain PR proteins (Morkunas and Ratajczak 2014; Kanwar and Jha 2019). For example, the hemibiotrophic maize pathogen *Zymoseptoria tritici* is known to express genes related to detoxification of phytoalexins at the early stage of infection of a susceptible host (Zhong et al. 2020). Likewise, it has recently been reported that *T. destructans*, also a hemibiotrophic pathogen, has the potential to secrete proteins associated with detoxification in-vitro in addition to fungal effector proteins, peptidases, kinases, toxins and lipases (Havenga et al. 2021a, b). This suppression of host defense responses is relevant for biotrophic pathogens, throughout their life cycle, to avoid being killed or inhibited by host defense responses (Ferreira et al. 2006).

The enrichment analysis showed that TF’s *NAC*, *MYB, bHLH, ERF C2H2, WRKY,* and *bZIP* were up-regulated in *Eucalyptus* leaves mainly at the late stage of infection by *T. destructans*. However, a homologue of *ATAF1* (*NAC, Eucgr.F04341*) was up-regulated at both the early and late infection stages (Supplementary material Fig. S6). *ATAF1,* has been reported as a negative regulator of defense responses against fungal pathogens by suppressing SA signaling (Wang et al. 2009). Like our results (Swanepoel et al. 2021), showed that the expression of genes related to SA, JA, ABA and ET signaling were up-regulated at the early stage of infection in a resistant *Eucalyptus grandis* host infected by the biotrophic rust pathogen *Austropuccinia psidii*. The authors also showed that there was a significant lack of expression of these genes in the comparison with a susceptible host (Swanepoel et al. 2021).

The TF *MYB96,* a regulator of ABA signaling that induces flavonoid accumulation via the phenylpropanoid pathway (Zhang et al. 2021), was up-regulated at the early stage of infection and significantly down-regulated at the late stage. ABA treatment or overproduction is known to represses SA- and JA-regulated gene expression and reduce resistance to biotic stressors (Audenaert et al. 2002; Anderson et al. 2004; Yasuda et al. 2008; Aalto et al. 2012). However, it has recently been reported that ABA induces downstream elements that are similar to SA signaling outcomes to specifically promote stomatal closure (Du et al. 2024). Due to the potential role of ABA in regulating stomatal opening, there is a probable link to *T. destructans* biology where spores are known to infect and sporulate via the stomatal openings in the absence of cell disruption (Solís et al. 2022).

Genes involved in the flavonoid pathway were significantly enriched at the early and late stage of *T. destructans* infection. Catechin, quercetin and rutin showed significant differences between infected and control leaves. Rutin is the main glycosidic (a 3-O-rhamnoglucoside) form of quercetin in *Eucalyptus* and is also the most abundant flavonol in many vegetables and fruits (Lakhanpal and Rai 2007). Consequently, many plant-associated microbes are adapted to exist in the presence of this metabolite. For example, rutin promoted the germination of *Alternatia alternata* and *Fusarium solani* (Kalinova and Radova 2009) and plays an important role in the interaction between mycorrhizae and the roots of *Eucalyptus,* stimulating the hyphal growth of the mycorrhizal fungus *Pisolithus tinctorius* (Lagrange et al. 2001). Likewise, quercetin can be degraded by certain fungi and used as a carbon source. For example, *Sclerotinia sclerotiorum* possesses a quercetin dioxygenase gene which encodes a protein that catalyzes the cleavage of the flavonol carbon skeleton (Chen et al. 2019). Interestingly, in our study, both quercetin and its glyosidic form, rutin, promoted *T. destructans* germination in-vitro. Leaf ontogeny had a significant effect on flavonol accumulation and both compounds, but especially rutin, which was more abundant in the younger leaves. This is consistent with the fact that *T. destructans* commonly infects younger leaves and thus higher levels of flavonols could serve as recognition signals, stimulating spore germination.

The flavan-3-ol catechin, the monomeric precursor of condensed tannins, inhibited *T. destructans* germination in-vitro*.* The accumulation of catechin and its polymeric derivatives has been reported to be an effective defense response to pathogen infection. For example, a high accumulation of flavan-3-ols, inhibited rust spore germination and reduced hyphal growth in-vitro in the resistance of poplar to infection by *Melampsora larici-populina* (Ullah et al. 2017). Catechin was also reported to inhibit appressorium development of *Colletotrichum kahawae* the causal agent of coffee berry disease (Chen et al. 2006) and to inhibit several fungal enzymes (Prusky et al. 1988; Niu et al. 2004). In poplar leaves, catechin accumulates in the epidermal layers of resistant poplar genotypes (Ullah et al. 2017). In the present study, significantly lower concentrations of catechin at the early stage of infection in younger leaves might reflect a lack of such a defense barrier in susceptible *Eucalyptus* leaves.

The terpenoid 1,8-cineole (eucalyptol), inhibited *T. destructans* spore germination in-vitro, even at the lowest concentration tested. Volatiles, such as eucalyptol are secreted and stored as a constitutive defense barrier in glandular trichomes in *Eucalyptus* leaves (Heskes et al. 2012). However, external stimuli can trigger the de novo production and emission of terpenoids via plant defense hormones. For example, SA accumulation in *Populus nigra* infected by the rust fungus *M. larici-populina* induced terpene emissions from leaves as a defense response (Eberl et al. 2018). The mechanism underlying the antifungal activity of monoterpenes, such as 1,8-cineole have not been fully elucidated. However, some studies have reported that they are lipophilic agents that can solubilize membranes and interact with membrane embedded enzymes (Uribe et al. 1985; Sikkema et al. 1994; Prashar et al. 2003). For example, the mode of action of 1,8-cineole in inhibiting *Streptococcus pyogenes* is by affecting the cell-surface hydrophobicity, affecting auto-aggregation (biofilm formation) and extracellular proteases, as well as the expression of virulence genes (Vijayakumar et al. 2020). These modes of action could underly the antifungal activity of the metabolite in *T. destructans*, however the effect of this terpenoid on the pathogen’s cell membranes is not known.

The results of this study showed that *Eucalyptus* hosts with delayed and attenuated defense responses are susceptible to *T. destructans*. Host susceptibility appears to be characterized by a lack of early defense responses, a delayed SA-mediated signalling event during the pathogen’s necrotrophic phase and the synthesis of secondary metabolites that promote pathogen germination in-vitro. Future studies should aim to compare these responses in hosts with different levels of susceptibility and consider the role of phytohormones in regulating an appropriate response to pathogen infection. Future comparisons of both resistant and susceptible host responses would allow the identification of genes related to resistance and thus make it possible to select individuals with more durable resistance to *T. destructans*.

## Supplementary Information

Below is the link to the electronic supplementary material.

Supplementary file1 (DOCX 1299 kb)

## Data Availability

The authors confirm that all data generated from this study is available and can be found in this article and in the supplementary information.
