# Supplementary material for: Transcriptional responses of Eucalyptus to infection by an aggressive leaf blight pathogen reveal the role of host secondary metabolites during pathogen germination
Source: Plant Mol Biol. 2025 Aug 11;115(5):104. doi: 10.1007/s11103-025-01625-2 (PMC12339631; doi:10.1007/s11103-025-01625-2)
Supplement: Supplementary file 1 — Supplementary file1 (DOCX 1299 kb) [file 11103_2025_1625_MOESM1_ESM.docx]

**Supplementary Material**


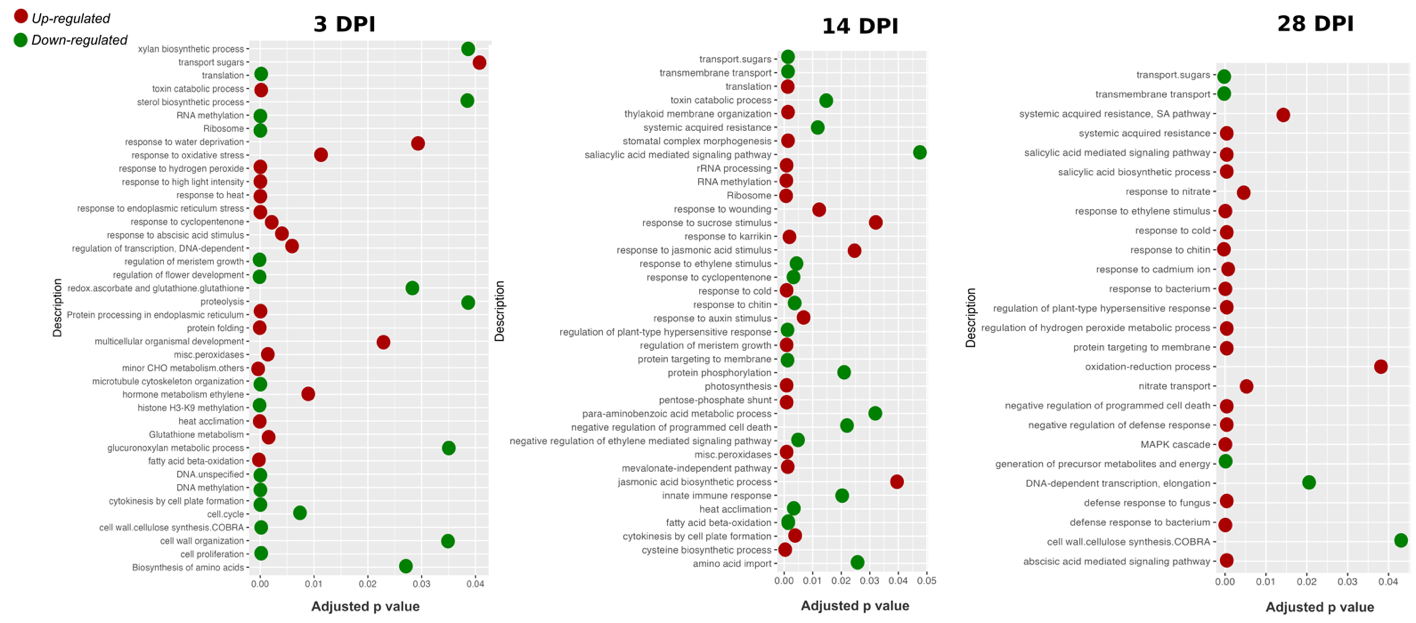


**Figure S1.** Kyoto Encyclopedia of Genes and Genomes (KEGG) analysis of significantly up-regulated and down-regulated differentially expressed genes (DEGs) over time showing the significance of each over-represented term. Colored dots indicate significance using adjusted *p-value <* 0.05.

**
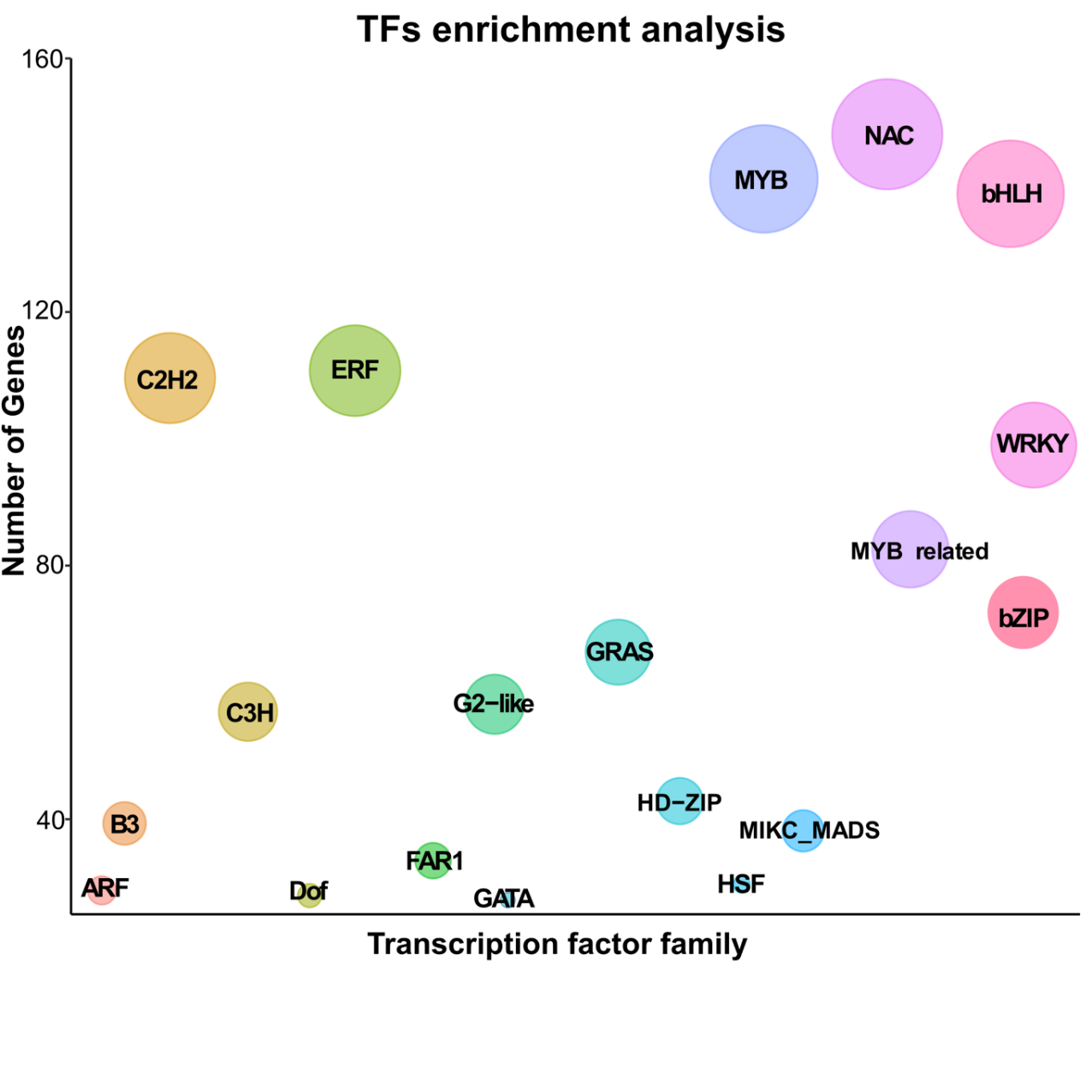
**

**Figure S2.** Transcription factor enrichment analysis across the three time points. Circles represent the number of significantly differentially expressed genes (DEGs). Colours indicate the transcription family annotated with Plant Transcription Factor Database v4.0.


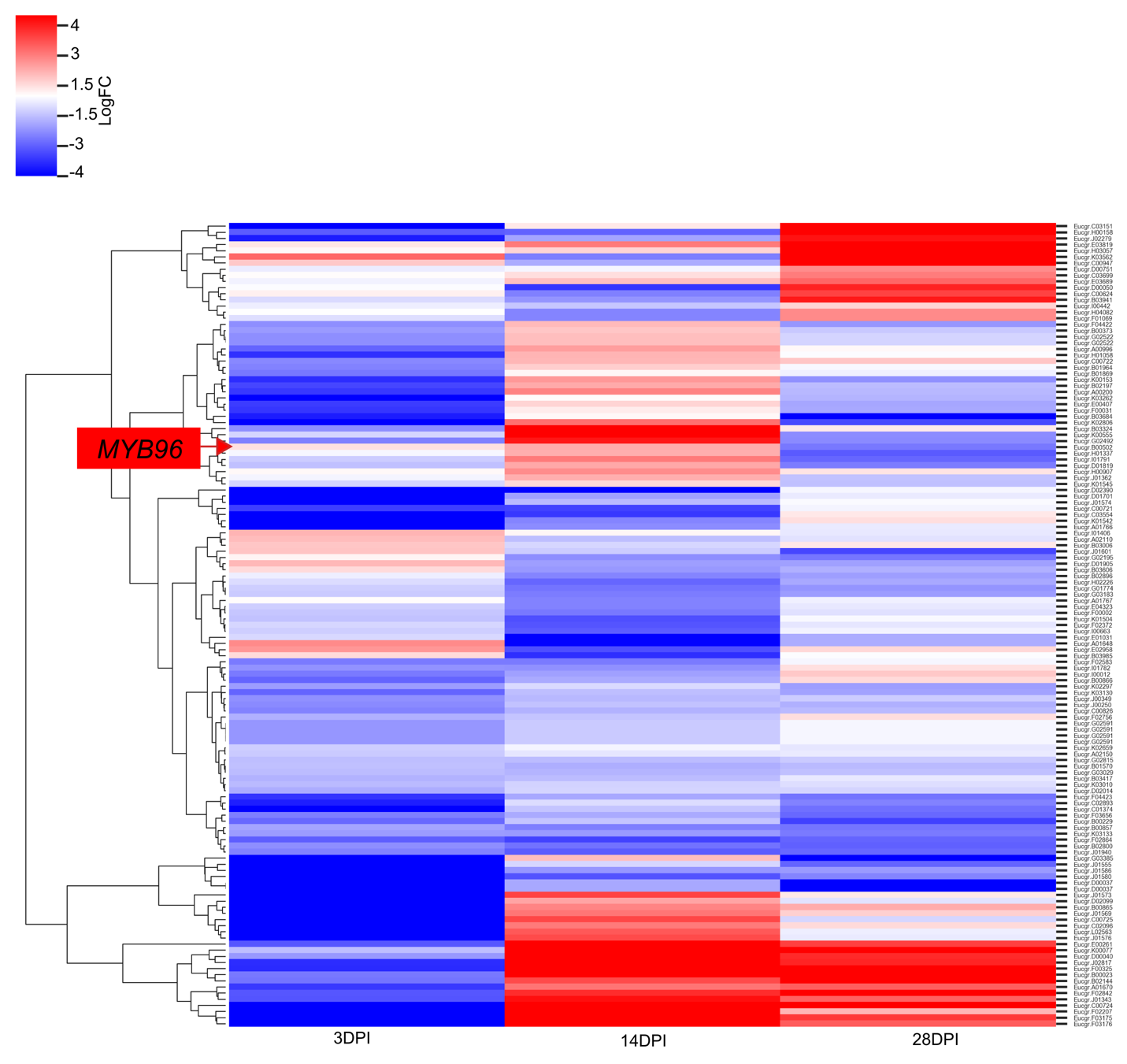


**Figure S3.** Heat map showing the expression of MYB transcription family of up- (red) and down-regulated (blue), differentially expressed genes at 3, 14 and 28 dpi (FDR adjusted *p*-value ≤.05). Red arrow indicates the early expression (3 dpi) of *Eucgr.B00502* homologue to *MYB96*, a major regulator of ABA hormone signaling.


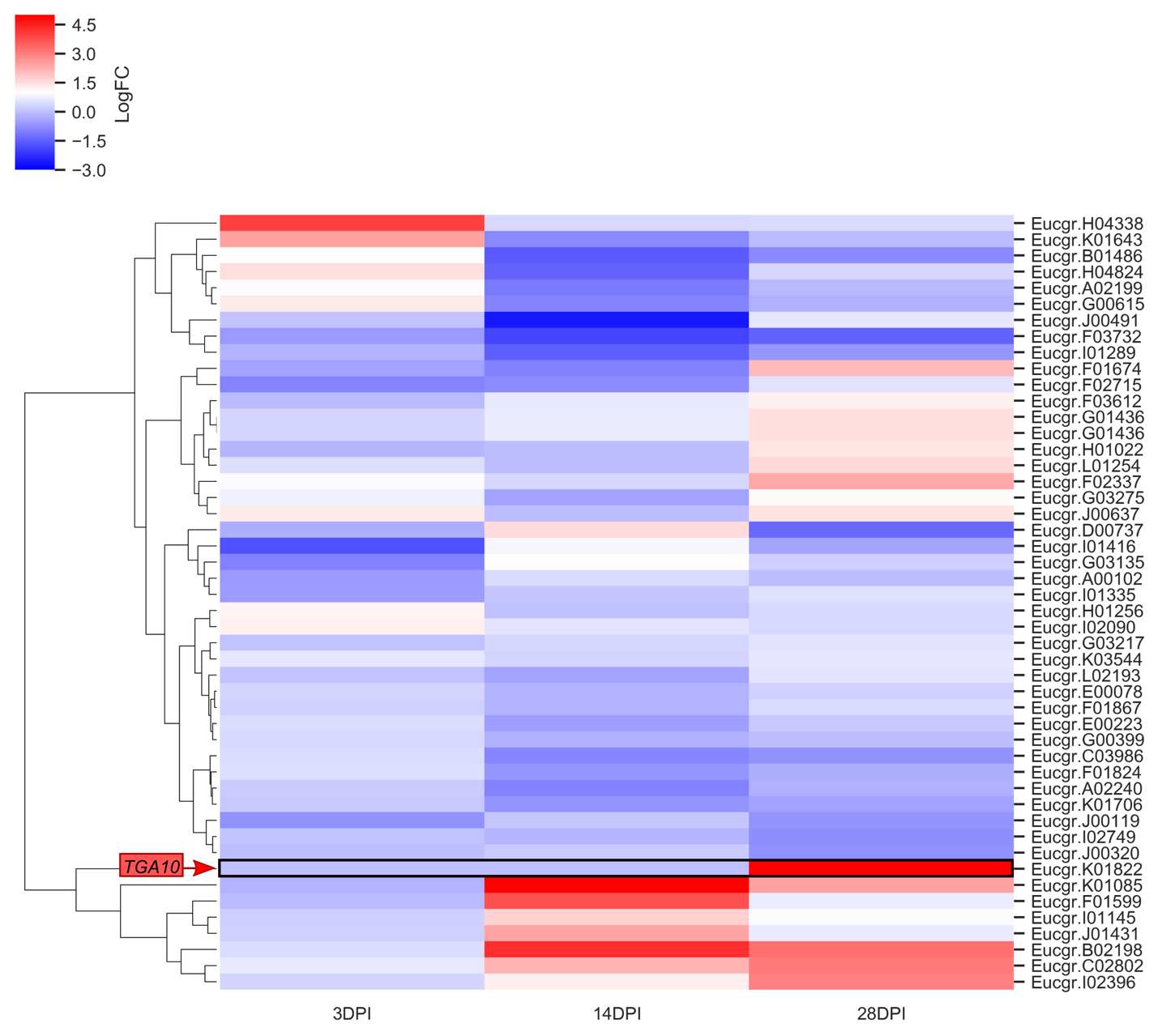


**Figure S4.** Heat map showing the expression of *bZIP/TAG* transcription family of up- (red) and down-regulated (blue), differentially expressed genes at 3, 14 and 28 dpi (FDR adjusted *p*-value ≤.05). Red arrow indicates the late expression (at 28 dpi) of *Eucgr.K01822* homologue to *TGA10* a major regulator of SA signaling.


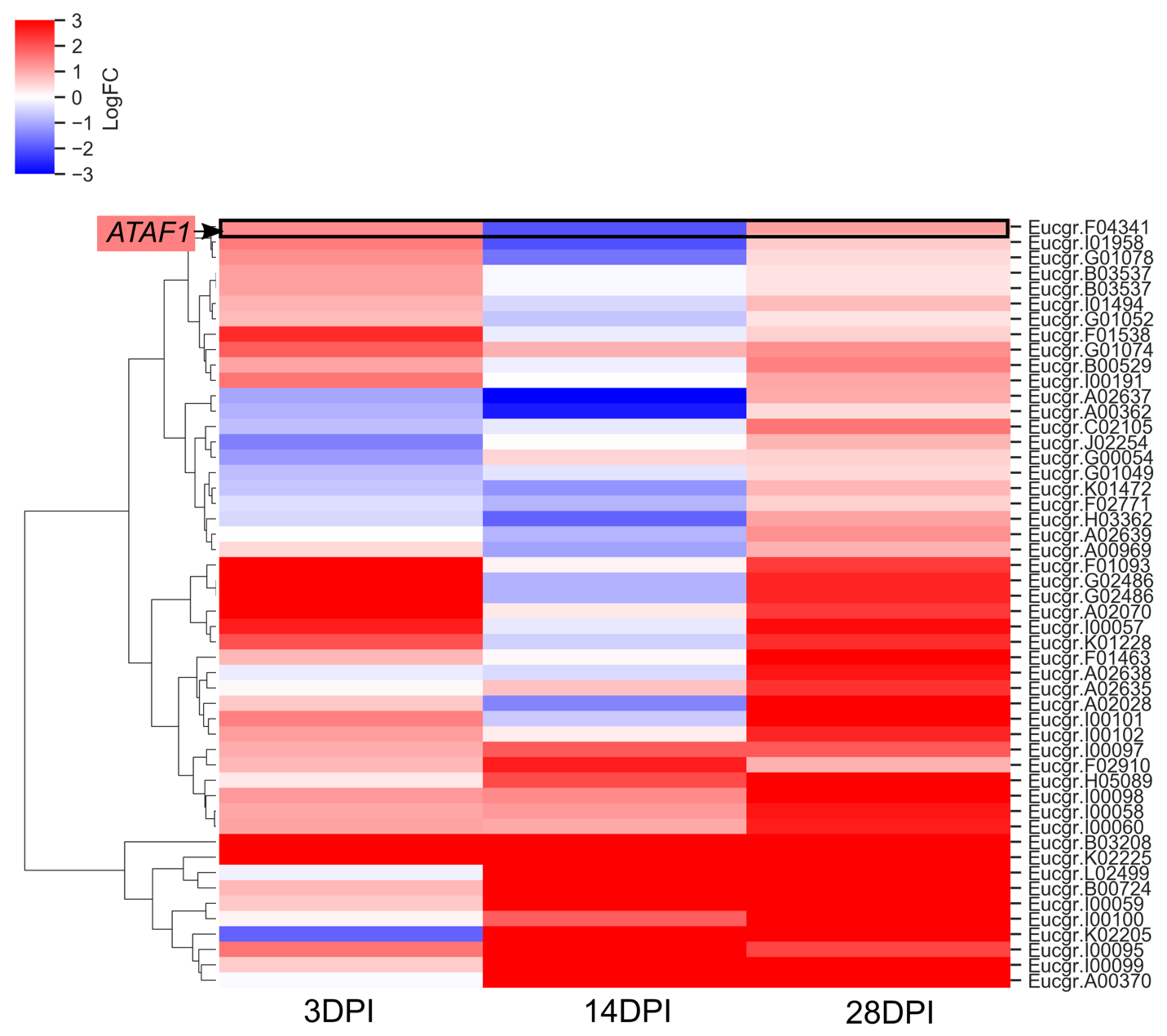


**Figure S5.** Heat map showing the expression of *NAC* transcription family of up- (red) and down-regulated (blue), differentially expressed genes at 3, 14 and 28dpi (FDR adjusted *p*-value ≤.05). Red arrow indicates the late expression (at 28 dpi) of *Eucgr.F04341* homologue to *ATAF1* a negative regulator of plant defense response.
